# Indoor Evaluation of a Temperature-Controlled Gel Intelligent Diversion System

**DOI:** 10.3390/nano15070547

**Published:** 2025-04-03

**Authors:** Zhifeng Luo, Qunlong Wu, Weiyu Chen, Haoran Fu, Kun Xu, Haojiang Xi

**Affiliations:** 1 School of Petroleum and Natural Gas Engineering, Southwest Petroleum University, Chengdu 610500, China; lzf103429@163.com (Z.L.); 19983451050@163.com (W.C.); 13399835448@163.com (H.F.); nineseven105@gmail.com (K.X.); xideea@gmail.com (H.X.); 2 State Key Laboratory of Oil and Gas Reservoir Geology and Exploitation, Southwest Petroleum University, Chengdu 610500, China

**Keywords:** reservoir heterogeneity, temporary plugging and flow diversion, reservoir transformation, uniform acidizing, temperature-controlled diversion system, indoor evaluation

## Abstract

The Bohai SZ36-1 oilfield, the largest offshore oilfield in China, features a high-porosity, high-permeability reservoir with significant heterogeneity and permeability variations. After extended water injection, the reservoir’s pore structure evolved, increasing heterogeneity and reducing the effectiveness of traditional production methods. To address these issues, this study introduces an intelligent diversion and balanced unblocking technology, using a temperature-controlled diversion system to block dominant flow channels and ensure even distribution of treatment fluids while maintaining reservoir integrity. The technology’s scientific validity and feasibility were confirmed through extensive testing. Results show that the diversion system offers excellent injectability, with controllable solidification time, phase change temperature, and strong compatibility, allowing for a “liquid–solid–liquid” phase transition in the reservoir. The technology also demonstrates high plugging strength, rapid plugging rate, significant diversion effects, and moderate injection intensity, all meeting construction requirements.

## 1. Introduction

Temporary plugging and diversion acidizing [1,2] is a key technology for enhancing oil well production and facilitating reservoir transformation. It is particularly effective for sandstone reservoirs, exhibiting strong vertical heterogeneity, prominent dominant flow channels, low residual oil recovery in secondary channels, and the presence of numerous small layers and interlayers [3,4,5]. Once introduced, the diverter temporarily blocks the dominant flow channels, forcing the subsequent treatment fluids (e.g., acid) to be redirected into the secondary channels [6,7,8,9]. This diversion ensures uniform distribution of the working fluid, leading to more efficient mobilization of the formation surrounding the well and ultimately improving the oil recovery rate [10,11].

Currently, temporary plugging and diversion techniques are primarily classified into two categories: physical diversion and chemical diversion. Physical diversion, also known as external diversion, typically occurs within the wellbore. It involves isolating or plugging specific sections of the formation to control the flow of fluids into the well, thereby achieving diversion [12,13,14]. Common physical diversion methods include packer diversion [14,15], coiled tubing diversion [16,17], and particle diversion [18,19]. In contrast, chemical diversion, or internal diversion, occurs within the formation itself by increasing the resistance to fluid flow [11]. Typical chemical diversion methods include foam diversion [20,21], hydrophobic aggregate diversion [22], and VES-based diversion acid technologies [23,24,25]. Chemical diversion is generally more efficient than physical diversion. While physical diversion demands advanced technology, equipment, and complex procedures, chemical diversion can be carried out within the reservoir itself. However, the effectiveness of chemical diversion is often constrained by the availability and suitability of diversion materials, which can make achieving truly efficient diversion challenging.

In recent years, VES (Viscoelastic Surfactant) steering acid technology has gained widespread use in reservoir stimulation. This technology not only causes less damage to the formation but is also easy to manage and retrieve, and its surfactants can be optimized to enhance diversion acidizing efficiency. In 2019, Mao et al. [26] synthesized four ammonium-based salt surfactants derived from erucic acid amide. Their comparative analysis revealed that barium-based surfactants generally have a lesser impact on rock wettability. In a separate study in 2018, Zhang et al. [5] observed that the adsorption of cationic surfactants onto negatively charged minerals negatively impacts permeability. Han et al. [27] also confirmed this finding, adding that this issue can be mitigated by using amphoteric surfactants, which are electrically neutral.

The Bohai Oilfield reservoir is characterized by high porosity and permeability, significant vertical permeability variations, high water cut, strong heterogeneity, and interlayers of varying thickness with well-defined dominant flow channels [28,29]. These factors make it challenging to distribute the working fluid evenly, complicating the blockage removal process. Given these reservoir characteristics, this paper proposes an innovative approach for intelligent diversion and balanced blockage removal in heterogeneous reservoirs. The method utilizes the amphiphilic associating molecule TDS (the system exhibits excellent temperature responsiveness) in combination with the concept of VES steering acidization. An indoor evaluation of this technology is also conducted.

## 2. Experiments

### 2.1. Materials

**Figure 1:**
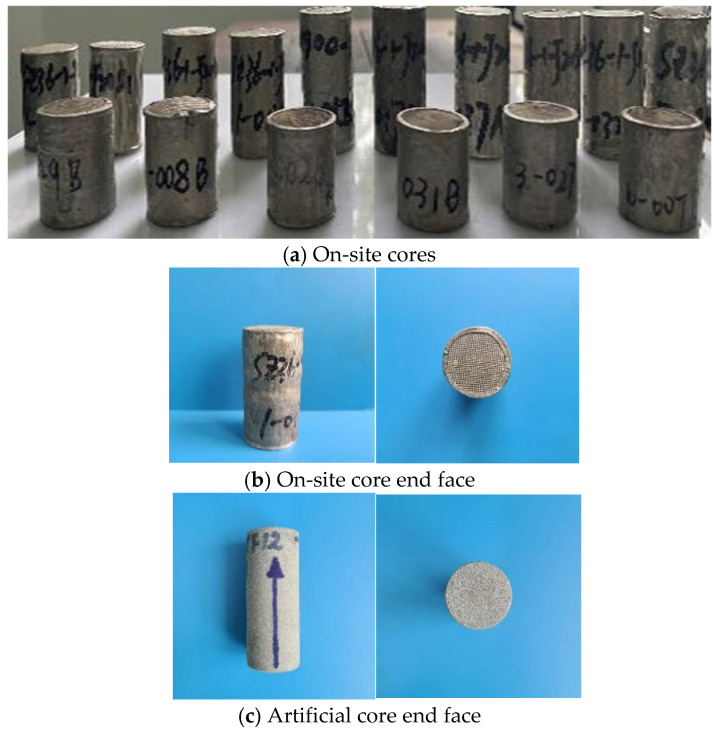
Core samples.

The experiment utilized both on-site cores from the Bohai Oilfield and artificial cores for testing. The on-site cores were employed for parallel flow displacement experiments, while the artificial cores were used to evaluate the plugging rate and plugging strength. In accordance with the SY/T 6385-2016 [30], the core length ranges from 2.5 to 5.0 cm, with a diameter of approximately 2.5 cm. The core’s basic parameters are presented in Table 1, and a visual representation of the core sample is shown in Figure 1.

**Figure 2:**
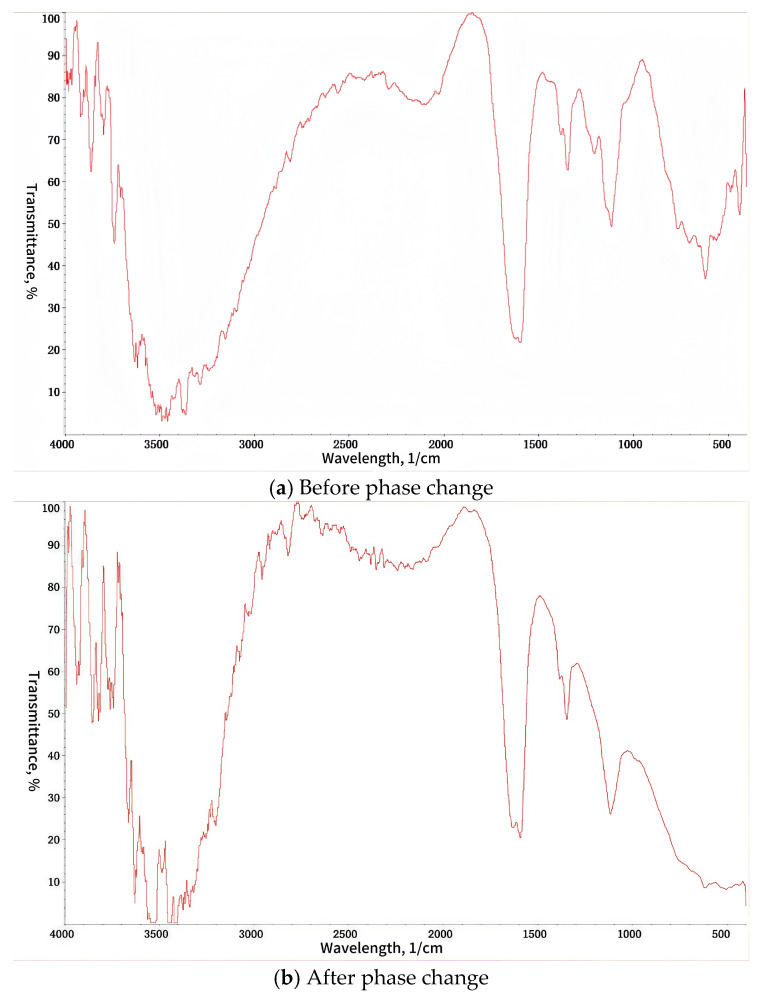
FT–IR spectrum of TDS.

**Figure 3:**
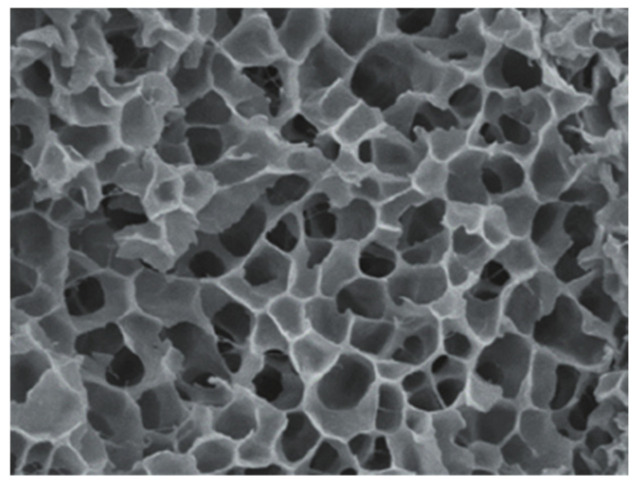
Microstructure of TDS.

**Figure 4:**
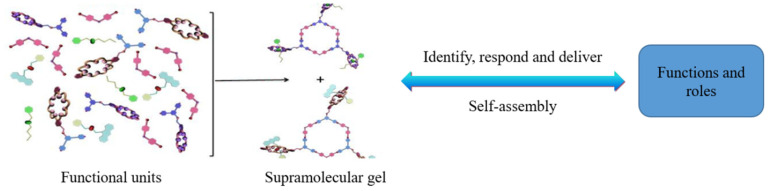
Construction of TDS.

The key to the intelligent diversion and balanced plugging removal technology of heterogeneous reservoirs lies in a new temperature-controlled diversion system. The system is based on the principle of thermo-induced supramolecular gel developed by Zhou et al. [31] and uses COF [32] molecules (association molecule forming agent PK01), which have characteristics of hydrophobic inner cavity, hydrophilic outer cavity, rich hydrogen bonds, good reversible saturation, strong guest molecule release ability, and good biocompatibility. The system is modified by adding additives, such as phase change promoter (PK02), modifiers, such as PO4 (including iron ion stabilizer, corrosion inhibitor, coagulant, scale inhibitor), and other guest molecules in order to enable it to respond to various temperature conditions and adapt to the requirements of different target reservoir temperatures. Next, through “retrosynthetic analysis” and “molecular dynamics” simulation, special functional units are designed and synthesized, and then the monomer structure is confirmed by infrared spectroscopy, SEM (scanning electron microscope) and other technologies (as shown in Figure 2 and Figure 3), and finally assembled into TDS. A schematic diagram of the construction of TDS is shown in Figure 4.

**Figure 5:**
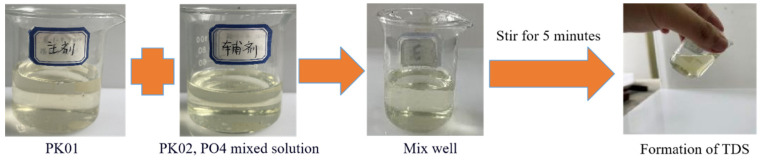
Preparation of TDS.

The formulation of TDS consists of PK01, PK02, the auxiliary agent PO4, and an organic solvent. The concentration ranges are as follows: PK01 at 1.5–6%, PK02 at 0.1–0.5%, PO4 at 4–7%, and the regulator at 0.15–1%, with the remainder being organic solvent. The concentration of each component is adjusted based on the temperature conditions of the target reservoir and the duration of the process. The system configuration is shown in Figure 5. The main agent in the figure is PK01, and the auxiliary agent is a mixed solution of PK02 and PO4.

**Figure 6:**
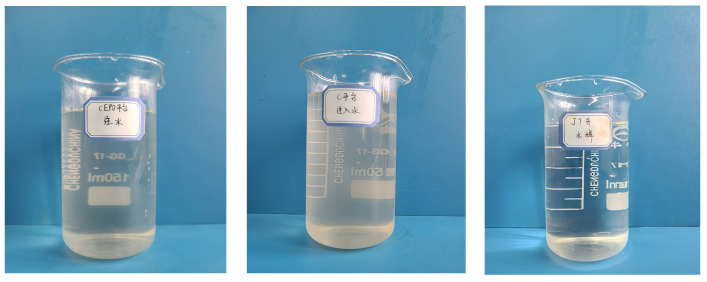
Formation water samples.

The formation water samples used in the experiment were collected from the Bohai Oilfield, including injection water from the CEPO platform, the C platform, and the J7 well. The first two water samples were slightly turbid, while the J7 well water sample was clear and transparent. All samples had a faint rust odor. The formation water samples are shown in Figure 6. Figure 6 shows, from left to right, the injected water of CEPO platform, the injected water of C platform, and the formation water of J7 well.

### 2.2. Technical Mechanism

**Figure 7:**
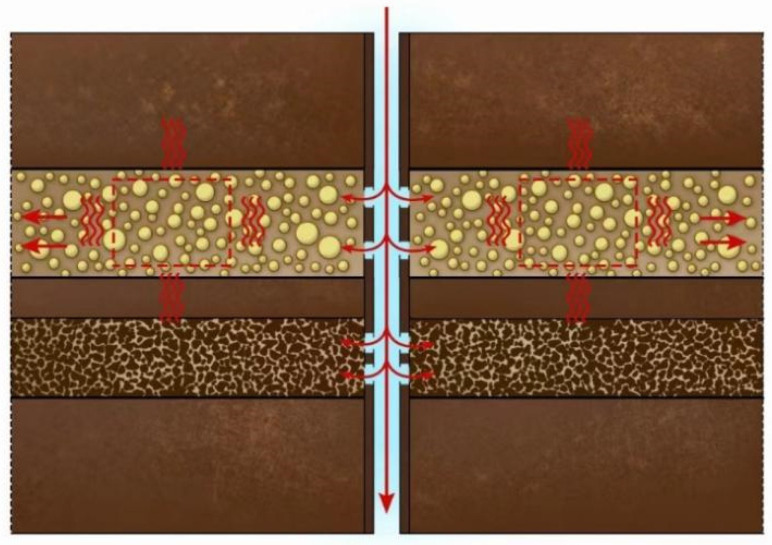
The mechanism of action of IDBPRT.

The working principle of the Intelligent Diversion and Balanced Plugging Removal Technology (hereinafter referred to as IDBPRT) for heterogeneous reservoirs is illustrated in Figure 7. The temperature-controlled diversion system (TDS) remains as a low-viscosity liquid in the subsurface environment, offering excellent injectability and stable properties. It undergoes a ‘liquid–solid–liquid’ phase transition under the influence of the reservoir’s natural temperature. According to the principle of minimum flow resistance, when the low-viscosity TDS enters the formation, it will first flow into the high-permeability zones. Once it reaches the target area, the formation temperature induces a phase change, transforming the TDS into a solid state. This creates plugging pressure that blocks the high-permeability reservoirs. Meanwhile, the subsequent working fluid is diverted into the low-permeability zones, ensuring uniform fluid distribution. After a set period, as the reservoir temperature increases, the solid diversion system thermally degrades, allowing the high-permeability zone to remain plugged, while the liquefied diversion system returns to the surface with the following working fluid.

**Figure 8:**
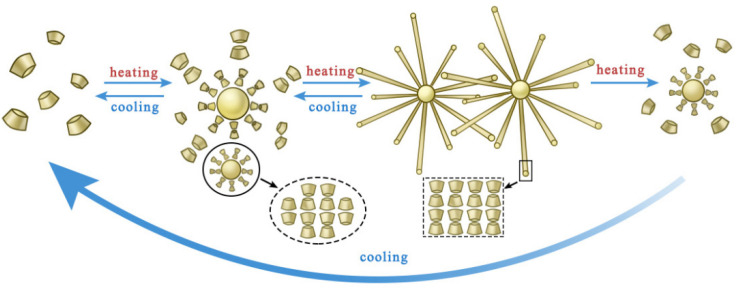
Liquid–solid–liquid phase transition mechanism of TDS.

TDS exhibits excellent phase-change behavior under temperature stimulation. At room temperature, PK01 and guest molecules are mutually soluble in organic solvents, resulting in weak intermolecular forces, and the system exists as a clear solution. As the temperature rises, the hydrogen bonds within the PK01 molecules gradually break and are replaced by intermolecular hydrogen bonds. In the presence of additives and regulators, these molecules begin to aggregate, forming cage-like particles and PK01 dimers. As the temperature continues to increase and approaches the ‘$T_{\mathrm{gel}}$’ point, a significant number of cage-like particles and PK01 dimers accumulate within the system. Upon reaching the ‘$T_{\mathrm{gel}}$’ temperature ($T_{\mathrm{gel}}$ is the gel-forming temperature), guest molecules and intermolecular forces trigger the formation of an ordered structure in PK01, with PK02 being encapsulated within the molecular clusters. This results in the entire system forming a gel with a certain degree of strength. When the temperature is further raised, the hydrogen bonds between the PK01 molecules within the gel break, causing the gel structure to collapse and restoring the system’s fluidity. A schematic diagram illustrating the phase change mechanism is shown in Figure 8.

### 2.3. Experimental Equipment

**Figure 9:**
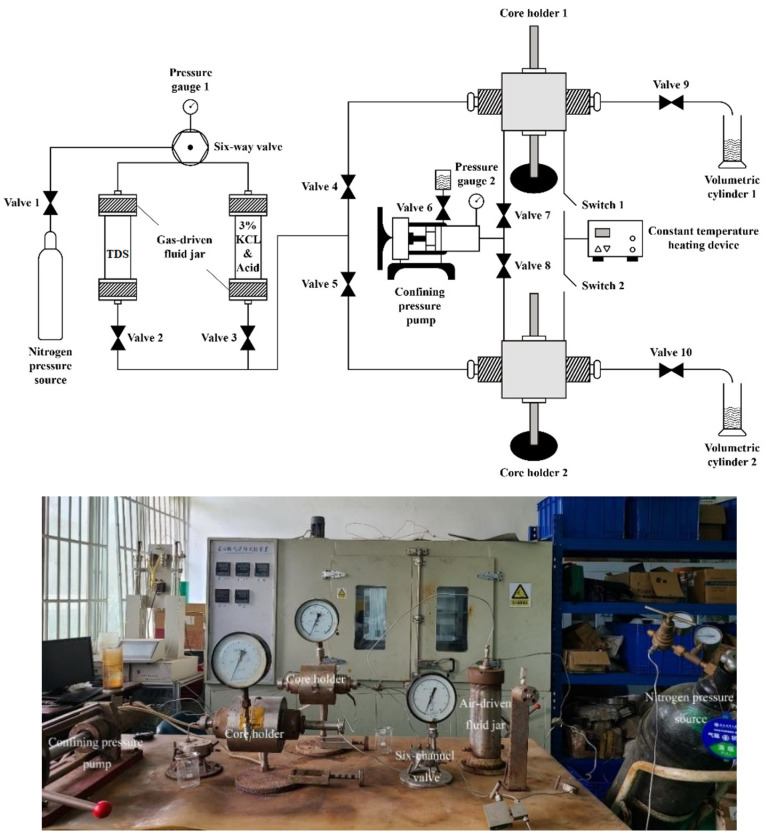
Parallel flow displacement device.

The study employed a parallel flow displacement device for indoor simulation to visualize the mechanism of action of the intelligent diversion and balanced blockage removal technology in heterogeneous reservoirs. This included the processes of injection, plugging, blockage removal, plugging release, and flowback. The device [33] consists of a nitrogen pressure source, a six-way valve, a gas-displacement liquid tank, a core holder, a confining pressure pump, a constant temperature heating device, a pressure gauge, and a measuring cylinder. The six-way valve controls the direction of the displacement pressure, while the gas-displacement liquid tank stores and replaces various fluids. The core holder is designed to clamp the core and apply pressure around it. This setup allows for simulating the temperature and pressure conditions of the target reservoir, enabling the temporary plugging and diversion of multi-level permeability reservoirs. Additionally, during single-channel experiments, the device can be used to test the plugging strength and plugging rate of the diversion agent. A schematic diagram of the device is shown in Figure 9.

**Figure 10:**
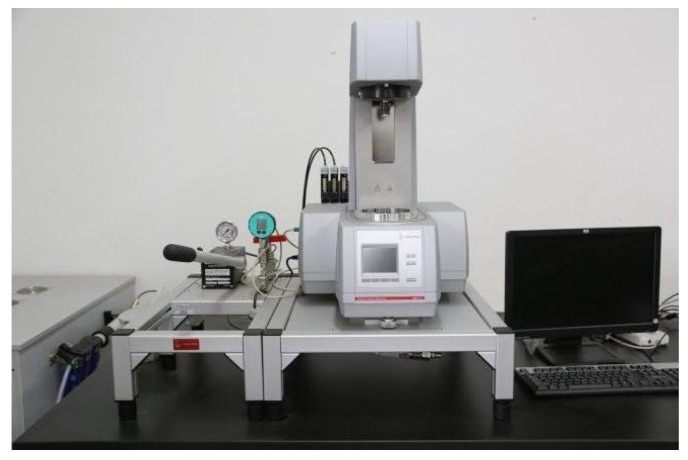
HAAKE MARSⅢ rotational rheometer.

The viscosity–temperature characteristics of TDS were measured using the HAAKE MARSⅢ rotational rheometer, as shown in Figure 10.

### 2.4. Experimental Methods

To prevent secondary damage, ensuring compatibility of the injected fluid is essential. We mixed TDS with formation water (including injection water from the C and CEPO platforms, as well as a C7 well water sample), kerosene, and acid (including hydrochloric acid and high-efficiency scale-inhibiting acid) in a 1:1 ratio. The mixture was then heated in a 60 °C water bath for 15 min, and we observed whether any precipitation or flocculants were formed.

Viscosity reflects the intermolecular interactions within a fluid and serves as a measure of its resistance to flow. To better understand the flow characteristics of TDS in wellbores and reservoirs, we used the HAAKE MARSⅢ rotational rheometer to measure the viscosity–temperature behavior of TDS. An appropriate amount of TDS was prepared and placed in a 500 mL wide-mouth bottle, from which air was evacuated before sealing. The sample was then placed in the HAAKE MARSⅢ rotational rheometer. The temperature range was set from 30 to 58 °C, the shear rate was 10 s⁻¹, and the heating rate was 1 °C/min. The viscosity change of TDS with temperature was subsequently measured.

The quality of the phase change characteristics is crucial for the efficient plugging and uniform distribution achieved by IDBPRT. A specific volume of TDS was placed in a 60 °C water bath and heated. The system’s state was recorded every three minutes until the TDS completed the liquid–solid–liquid phase change process, and the morphological characteristics at each stage were observed.

Intelligent flow distribution shows that the plugging time and temperature can be controlled. The auxiliary agent concentration of PK02 (hereinafter referred to as AAC), the ratio of PK02 to PO4 concentration and temperature are the main factors affecting the TDS curing time and temperature. Therefore, we adjusted the concentration of PK02 (3–9%), the ratio of PK02 (adding 0.3% or not adding) to PO4 concentration (2–7%) and the temperature (40–90 °C) to explore the rules of curing time and curing temperature.

After the preliminary material evaluation was completed, we conducted diversion experiments on four groups of cores with varying permeability ratios using the parallel core flow test device. The permeability ratios of the cores were 25 (core 1 and core 2), 16 (core 3 and core 4), 6 (core 5 and core 6), and 2 (core 7 and core 8), to simulate the action process of TDS in the formation. The experimental procedure was as follows:

1. Preparation: Prepare 50 mL of TDS and 1000 mL of a 3% KCl solution (with dye, hereinafter referred to as the base solution).
2. Core Setup: Place a group of cores in the core holder and apply a confining pressure slightly higher than the displacement pressure to prevent the cores from slipping during the displacement process.
3. Pre-washing: Inject the base solution to pre-wash the cores and record the flow rates of the two cores after the pressure stabilizes.
4. Injection of TDS: Inject TDS into the system and record the pressure and flow. When colorless droplets appear at the outlet, close the nitrogen bottle, open the vent valve, and release the pressure.
5. Core Heating: Heat the cores to a constant temperature of 70 °C for 45 min to allow the TDS to fully solidify.
6. Injection of Acid: Inject acid into the core and record the pressure and flow rates.
7. Degradation of TDS: Set the temperature to 85 °C and heat the system for another 40 min to degrade the TDS.
8. Final Injection: Inject the base fluid again and record the pressure and flow rates.

At the end of the test, the Darcy formula was used to analyze the pressure and flow rate data, which allowed us to determine the permeability changes before plugging, during TDS injection, after plugging, and after plugging was released. Finally, Origin software was used to analyze the diversion effect.

Plugging rate is a key parameter in evaluating the plugging performance of a diversion system. A higher plugging rate indicates better plugging effectiveness. Flow displacement experiments were conducted on five cores with permeabilities of 535.72 mD, 143.9 mD, 41.34 mD, 139.22 mD, and 256.03 mD, respectively. First, the cores were pre-flushed with the base fluid, and the flow rate at the outlet was measured once the pressure had stabilized. The initial permeability (K_1_) was calculated using Darcy’s formula. TDS was then injected into the cores and heated to allow curing. Afterward, the base fluid was injected again, and the pressure and flow rates were recorded to determine the permeability of the cores after plugging (K_2_). The plugging rate (w) was calculated based on the change in core permeability before and after plugging, as shown in the following equation:

(1) $$w=(K_{1}-K_{2})/K_{1}\times 100\%$$

The effectiveness of diversion is not only closely related to the plugging rate but also to the plugging strength. Breakthrough pressure is a key indicator used to characterize plugging strength. To assess whether the clean and intelligent diversion technology provides sufficient plugging strength, static breakthrough pressure tests were conducted on three cores. The procedure was as follows:

1. The core was placed in the holder and secured with a confining pressure (as in the core parallel flow experiment).
2. The core was pre-flushed with the base fluid.
3. TDS was injected into the cores and heated to allow curing.
4. The base fluid was injected using an advection pump to gradually increase the pressure.
5. The pressure and flow rate were monitored. When the flow rate suddenly increased, the corresponding pressure value at that moment was recorded as the static breakthrough pressure.

Injection strength is a key parameter for evaluating the feasibility of the process. Excessively high injection intensity may damage the reservoir, while too low an intensity could make the process difficult to implement. Based on the measured plugging strength data, we determined the temporary plugging length to be 0.3 m and used field well data for simulation research. The model is as follows:

(2) $$\frac{V}{h_{e}}=n\pi \alpha \text{∅}\left[{\left(L+L^{\prime }\right)}^{2}-r_{w}^{2}\right]$$

By inputting the basic parameters (see Table 2), setting the temporary plugging length to 0.3 m, and assuming a permeability of 5000 mD for the high-permeability reservoir, we varied the displacement (0.5 m^3^, 1.0 m^3^, 1.5 m^3^) and the maximum and minimum permeability ratios (5, 10, 20) to explore the injection intensity of the diversion system under different displacements.

## 3. Results

### 3.1. Characterization Analysis

There is a big difference in the absorption peak of TDS before and after the phase change. The peak of TDS is wider before the phase change, but there is no obvious absorption peak after the phase change. This shows that the curing process has a physical cross-linking effect. The change in the peak and the displacement of the peak are caused by the easy formation of hydrogen bonds between molecules during the gelation process of the TDS system. Compared with chemical cross-linking, physical cross-linking has the advantage of greater flexibility, thus ensuring that the TDS system can be liquefied to the greatest extent after gelation and restored to a solution state when heated.

It can be seen from the FT–IR spectrum that TDS has been reconstructed and the observed peak shift phenomenon is because the hydrogen bonding between O(3)-H and H-O(4′) and between C(7)-H and O-C(6′) inside the COF molecules disappears, and a large number of hydrogen bonds are generated between COF molecules, gradually transforming into cage-type particles.

According to the SEM scanning results, the molecules in the TDS gel system are arranged in an orderly manner and have a tight molecular structure, good stability and excellent mechanical properties.

### 3.2. Compatibility

**Figure 11:**
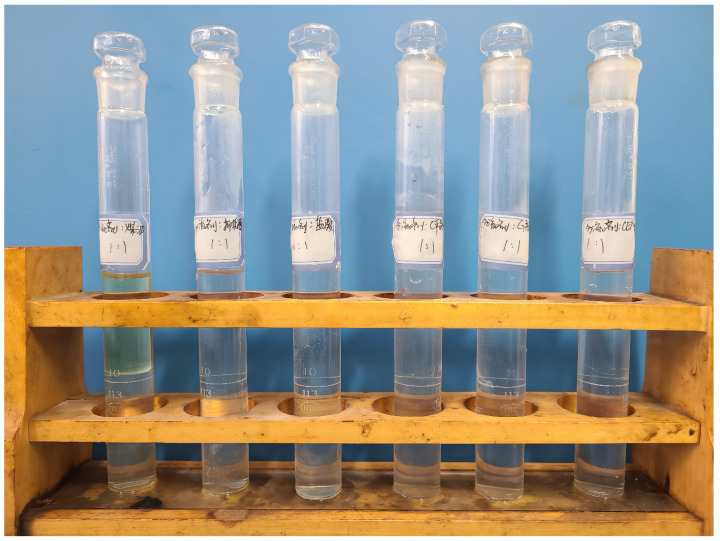
Results of compatibility test.

Figure 11 shows the results of the compatibility test. It can be seen that, after TDS was mixed with injection water, acid solution, and kerosene, no precipitation or flocculation occurred, indicating good compatibility. The stratification of the kerosene phase is a typical oil–water separation phenomenon. From left to right in Figure 11, the TDS of the split system is evenly mixed with kerosene, scale-inhibiting acid, hydrochloric acid, C platform injection water, J7 well formation water, and CEPO platform injection water in a 1:1 ratio.

### 3.3. Viscosity–Temperature Characteristics of TDS

**Figure 12:**
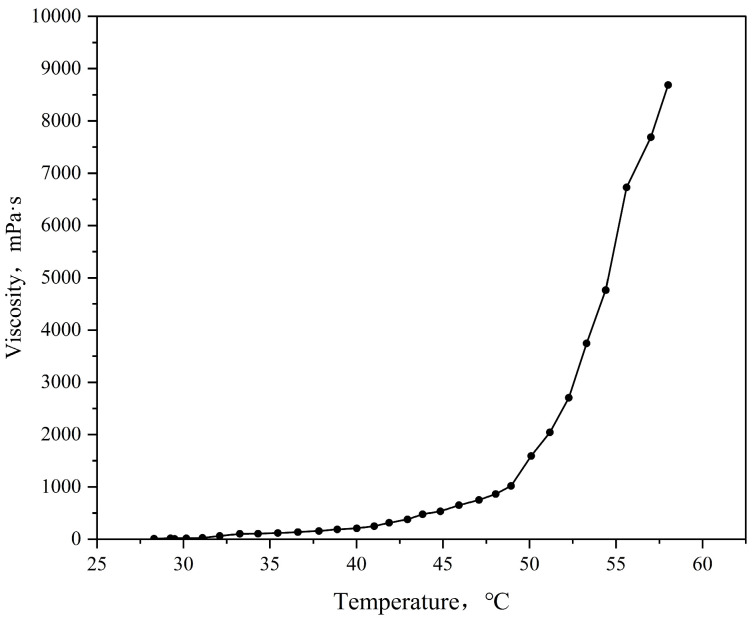
TDS viscosity–temperature characteristic test results.

The viscosity–temperature curve is shown in Figure 12. It can be seen from the figure that TDS is a low-viscosity fluid at room temperature with a viscosity of several millidarcy, which can last for nearly 20 min, providing enough time for the system to be transported to the target reservoir, effectively avoiding wellbore blockage, and having excellent injectivity. After reaching the target reservoir, the temperature reaches 55 to 60 °C, and TDS can quickly solidify into a solid gel to form an efficient plugging.

### 3.4. Phase Change Performance of TDS

**Figure 13:**
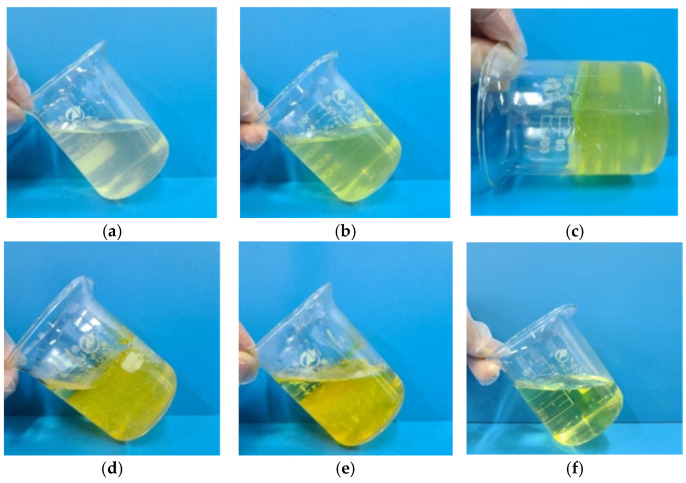
Phase change process of TDS (**a**–**f**).

The phase change process of TDS is illustrated in Figure 13. At room temperature, TDS is a low-viscosity liquid, maintaining a relatively clear appearance and exhibiting good fluidity. Upon heating to 70 °C for a certain period, the system’s color deepens, and viscosity increases gradually, while still retaining some fluidity. As heating continues, the viscosity increases rapidly, and the system solidifies into a high-strength polymer gel. Further heating causes the formation of internal pores within the gel, which progressively enlarge, with a small portion of the gel decomposing and liquefying. After extended heating, the gel continues to decompose, with the liquid phase increasing until it ultimately transforms into a clear yellow liquid. The results demonstrate that TDS can autonomously undergo a “liquid–solid–liquid” phase change under temperature stimulation, without the need for additional operations. This simple and straightforward process enables temporary plugging and diversion without damaging the reservoir. Once the plugging is released, the system returns to its liquid state, facilitating easy retrieval to the surface.

### 3.5. Curing Temperature and Curing Time

**Figure 14:**
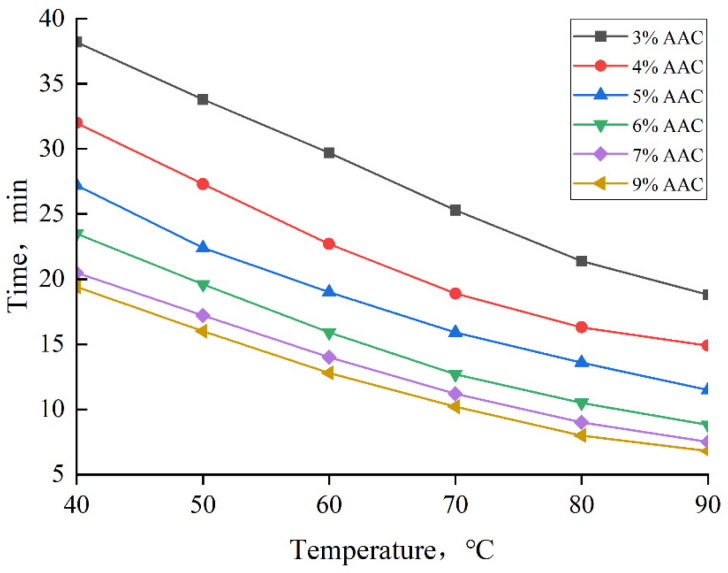
Curing time of TDS at different auxiliary agent concentrations and temperatures.

The relationship between TDS curing time, auxiliary agent concentration, and temperature is shown in Figure 14. The results indicate that TDS curing time can be intelligently controlled, with different concentrations of auxiliary agents chosen based on the reservoir temperature. At a constant auxiliary agent concentration, TDS curing time decreases as the temperature increases. Similarly, at a fixed temperature, the curing time decreases as the auxiliary agent concentration increases. However, the rate of decrease becomes less pronounced as the concentration rises. It is important to note that higher concentrations of the auxiliary agent do not necessarily lead to better results; rather, there exists an optimal concentration range of 4–7%. Beyond this range, increasing the concentration further has minimal impact on the curing time.

Table 3 presents the solidification state of TDS at different temperatures. Under constant conditions, the addition of PK02 lowers the solidification temperature of TDS, and increasing the concentration of P04 further reduces this temperature. Without PK02, as the concentration of P04 decreases from 4% to 2.5%, TDS solidifies between 80 °C and 90 °C. At lower temperatures, TDS can still solidify by appropriately increasing the concentration of P04. When PK02 is added, the concentration of P04 can be reduced from 7% to 4%, enabling complete solidification of TDS between 50 °C and 80 °C. At higher temperatures, TDS can also solidify effectively by adjusting the P04 concentration. These results demonstrate that the solidification temperature of TDS can be finely tuned by adjusting the concentration ratio of PK02 and P04. By selecting an appropriate TDS formulation based on the temperature environment of the target reservoir, the system’s solidification temperature can be precisely matched to the reservoir temperature, enabling efficient plugging and diversion without causing damage to the reservoir.

### 3.6. Parallel Flow Displacement Experiment

**Figure 15:**
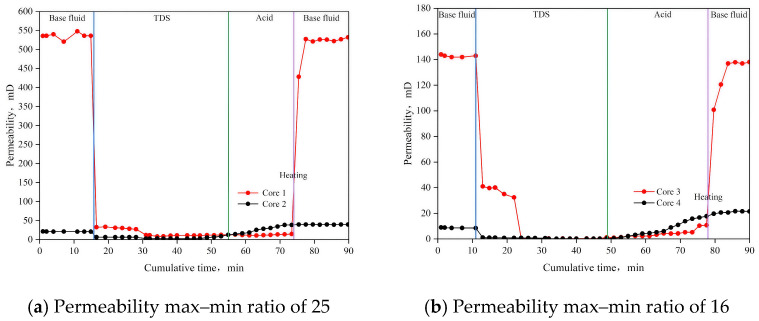

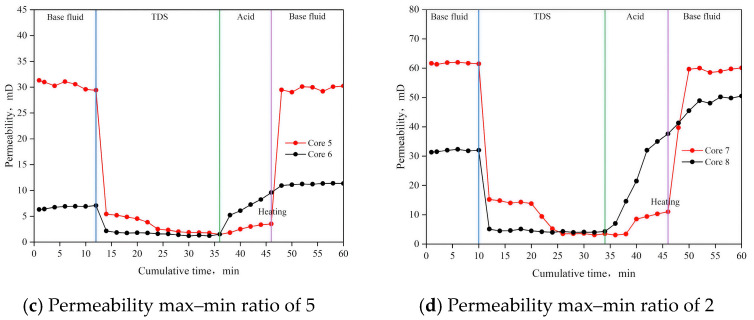
Results of parallel flow displacement experiments.

The results of the parallel flow displacement experiment are presented in Figure 15 and Table 4. The initial permeabilities of the high-permeability cores (1, 3, 5, and 7) were 535.72 mD, 143.90 mD, 31.34 mD, and 61.65 mD, respectively. After TDS plugging, the permeabilities decreased to 11.10 mD, 0.25 mD, 1.98 mD, and 6.57 mD, respectively. This significant reduction in permeability indicates that TDS is effective in achieving efficient plugging. For the low-permeability cores (2, 4, 6, and 8), the permeabilities after plugging were higher than those of the corresponding high-permeability cores, suggesting that the fluid flow in the high-permeability cores was diverted to the low-permeability cores, demonstrating a clear diversion effect. After the plugging was lifted, the permeabilities of the high-permeability cores recovered to 525.89 mD, 137.39 mD, 29.83 mD, and 59.50 mD, respectively, with recovery rates of 98.17%, 95.48%, 95.18%, and 96.51%. These recovery rates, all exceeding 95%, indicate that TDS is fully decomposed and can easily return to the surface, making it reservoir-friendly.

For the low-permeability cores, after diversion and unplugging, the permeabilities were 39.65 mD, 21.44 mD, 11.24 mD, and 49.49 mD, which were 1.85, 2.41, 1.78, and 1.58 times the initial permeabilities, respectively, showing a significant balanced diversion effect. After implementing this technology, the maximum and minimum permeability ratios were reduced to 13.4, 6.4, 2.6, and 1.2, respectively, indicating a substantial reduction in reservoir heterogeneity.

### 3.7. Plugging Rate and Plugging Strength

**Figure 16:**
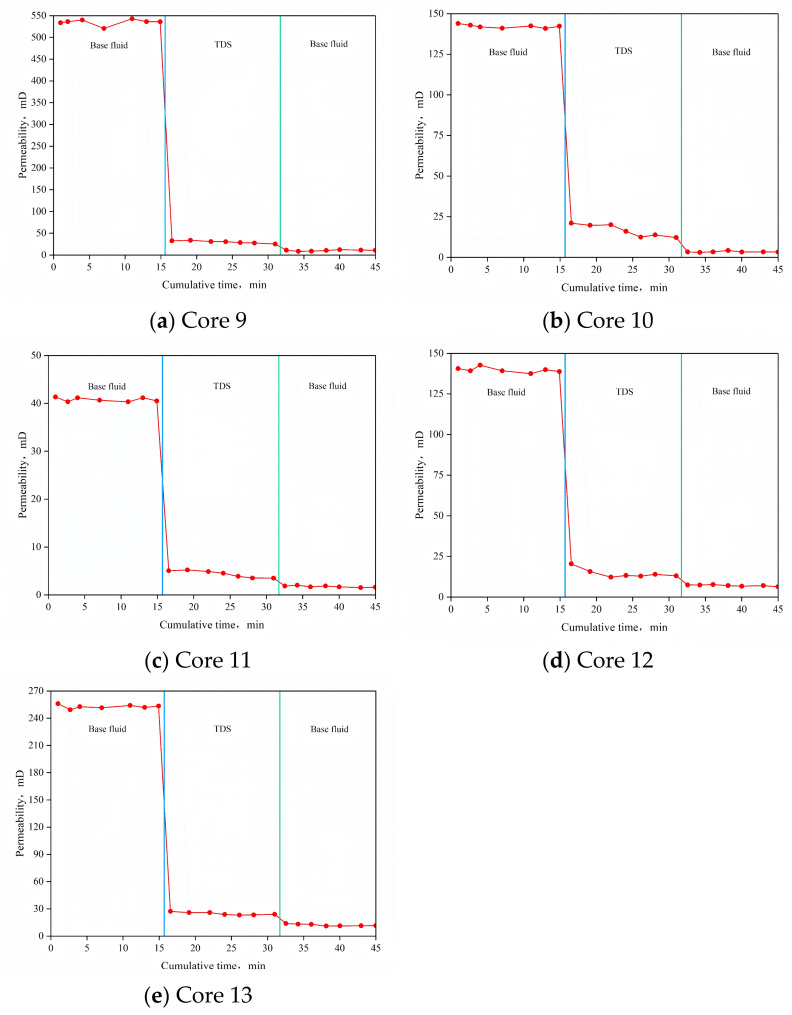
Results of the plugging rate test.

The results of the plugging rate test are shown in Figure 16 and Table 5. After TDS plugging, the permeability of each core was significantly reduced, with plugging rates of 98.05%, 97.62%, 95.73%, 95.00%, and 95.18%, all exceeding 95%. This indicates that TDS is highly effective in plugging the reservoir.

**Figure 17:**
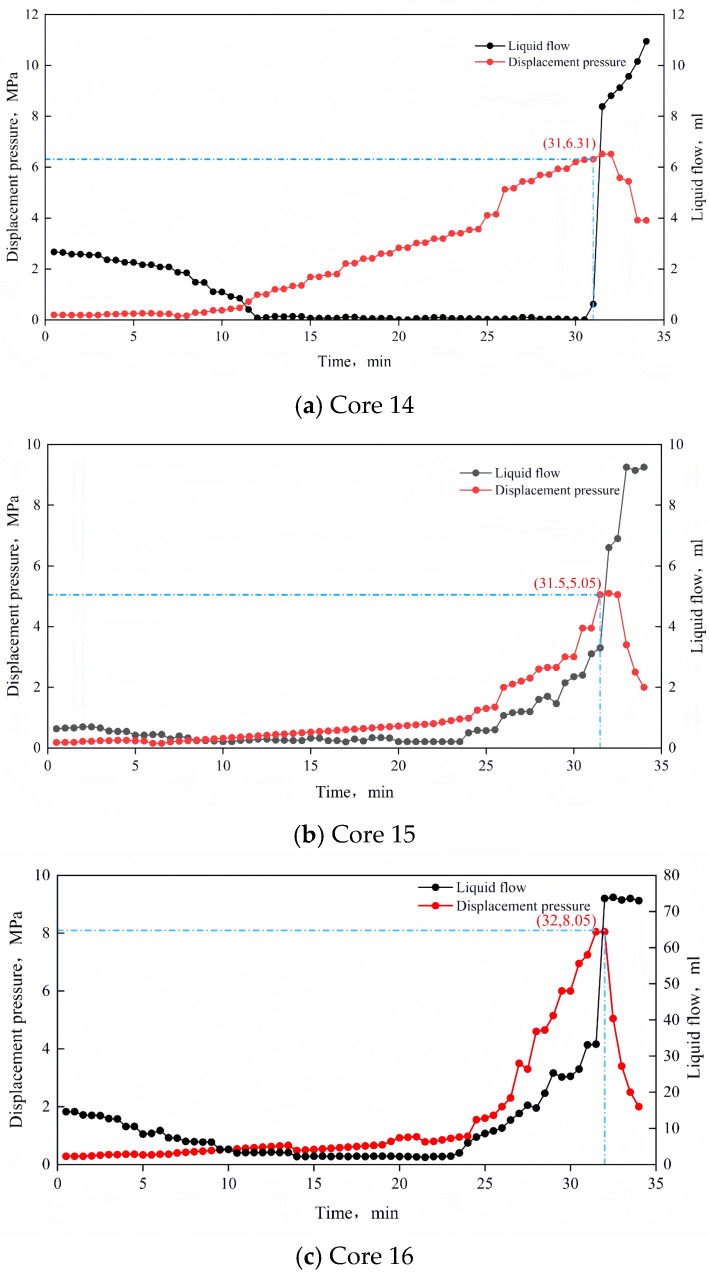
Test results of plugging strength.

The results of the static breakthrough pressure test are shown in Figure 17. The static breakthrough pressures for the three cores were 6.31 MPa, 5.05 MPa, and 8.05 MPa, respectively, all reaching several MPa. These values demonstrate that the plugging strength meets the required process standards.

### 3.8. Injection Strength

**Figure 18:**
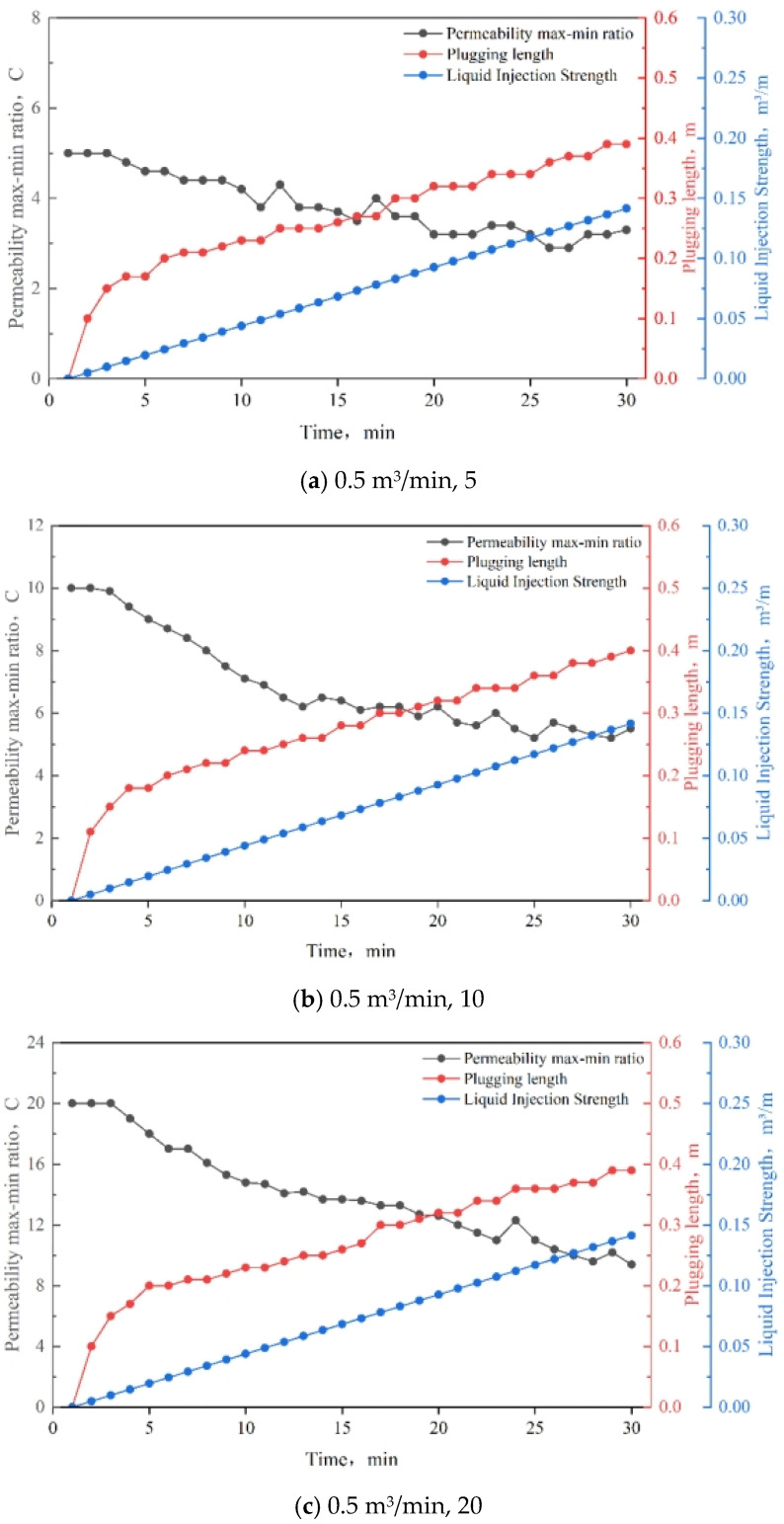

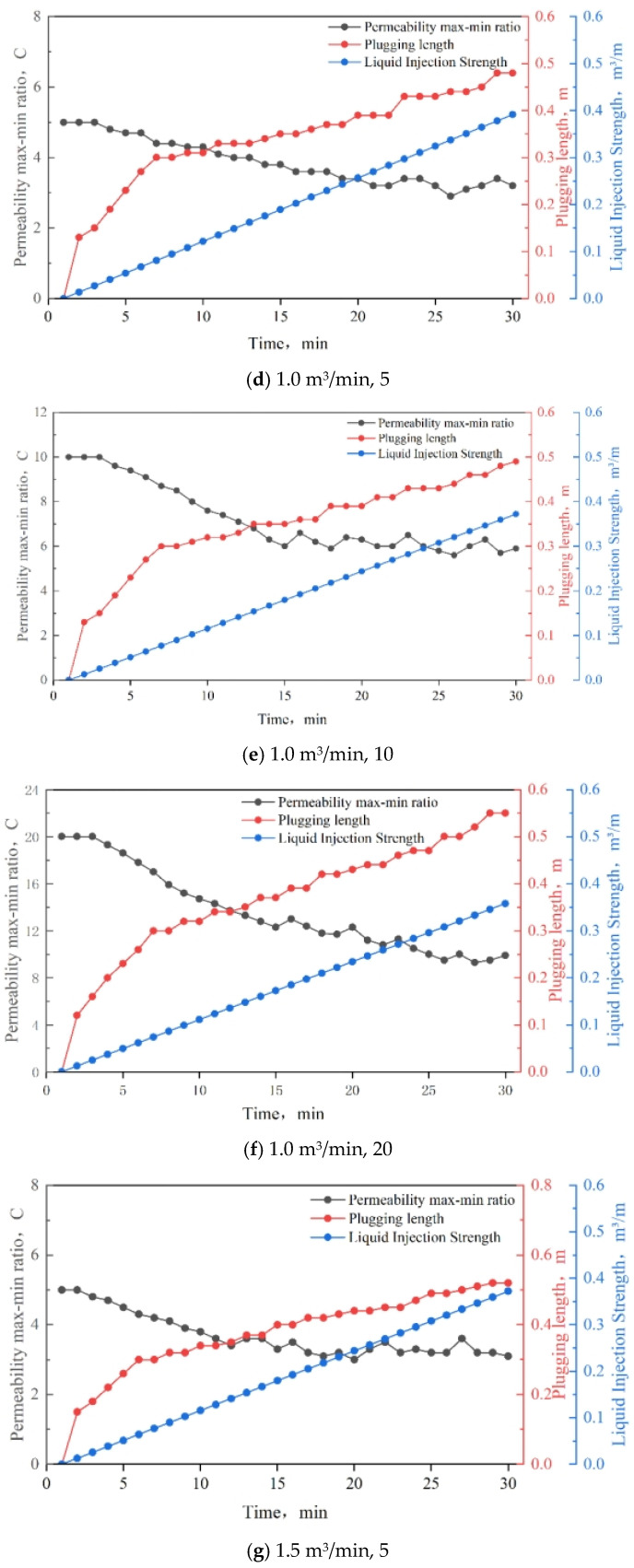

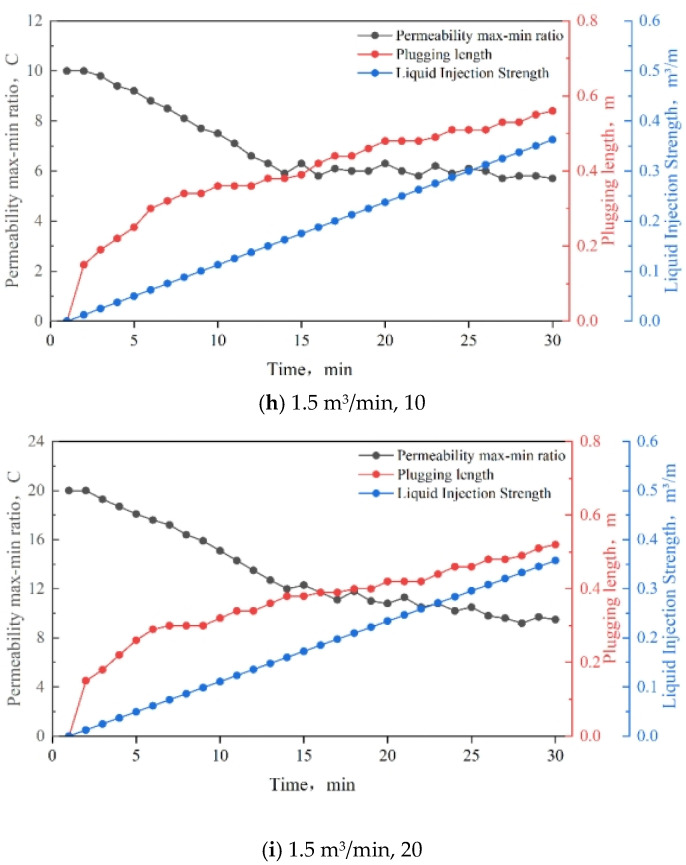
Results of the injection strength test.

The results of the injection strength tests are shown in Figure 18. It was observed that, as the displacement increases, the reduction in the permeability max–min ratio becomes smaller, indicating a less effective improvement in reservoir heterogeneity. To avoid phase changes in the wellbore, reducing the injection strength enhances the diversion effect. A graphical representation of the relationship between injection strength and the permeability max–min ratio is presented in Table 6. As shown in the table, the injection strength of TDS ranges from 0.074 to 0.083 m^3^/m, which is moderate and meets the construction requirements.

### 3.9. Slug Design and Application

TDS is injected as a slug by positive squeezing of the tubing, requiring only one or two TDS slugs to be added during the conventional acidification process. This approach simplifies the process and conserves platform space. The design details are shown in Table 6.

**Figure 19:**
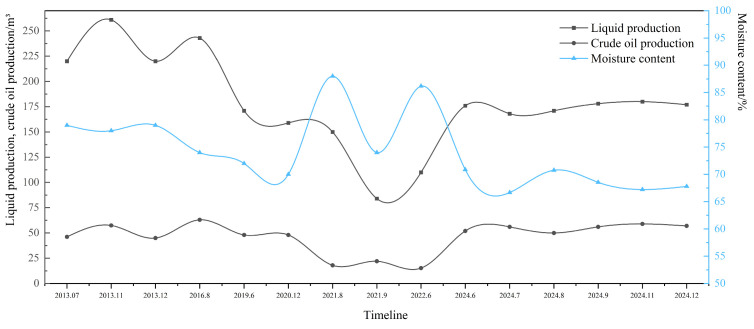
Statistics for trial production of SZ36-1-J1 well.

The temperature-controlled gel intelligent diversion technology was tested for half a year in the SZ36-1-J1 well in the Bohai Oilfield. Before the measures, the daily liquid production of the oil well was 110.24 m^3^, the daily oil production was 15.24 m^3^, and the water content was 86.17%. After the adoption of the temperature-controlled gel intelligent diversion technology, the acidification effect of the oil field was significantly improved, and the liquid production increased by 60% compared with before the measures, stabilizing at 175 m^3^/d; the crude oil content in the liquid production increased significantly, and the crude oil production increased by nearly 3.61 times compared with before the process, and can be stabilized at 55 m^3^/d; the water content of the output liquid was reduced by nearly 18% compared with before the process was implemented. See Figure 19 for details.

## 4. Conclusions

The temperature-controlled diversion system (TDS) demonstrates stable properties, excellent compatibility, superior injectability, strong temperature responsiveness, and efficient flow-back characteristics. It is an ideal material for enhancing production in heterogeneous reservoirs.

By adjusting the concentration of additives and auxiliary agents, the curing time and temperature of TDS can be intelligently controlled, allowing it to adapt to reservoirs with varying temperatures and properties. The system undergoes a liquid-to-solid-to-liquid phase transition under reservoir conditions and can automatically generate and degrade.

The system provides sufficient time for pumping to the target reservoir. Upon stimulation by the reservoir’s temperature, its viscosity increases rapidly, forming a high-strength gel. The resulting plugging strength exceeds 5.05 MPa, creating efficient sealing in high-permeability reservoirs, with a plugging efficiency of over 95%. This results in the redirection of flow from high-permeability zones to low-permeability zones, ensuring uniform acid distribution and effectively improving reservoir heterogeneity.

The injection intensity of the intelligent diversion and unblocking technology for heterogeneous reservoirs ranges from 0.074 to 0.083 m^3^/m, providing moderate strength that preserves the reservoir’s structural integrity while meeting the construction requirements.

The intelligent diversion and balanced plugging removal technology for heterogeneous reservoirs offers effective plugging, significant diversion capability, moderate injection intensity, and ease of operation. It requires only the installation of 1 to 2 TDS segment plugs in conventional acidizing procedures, without the need to modify the tubing. This technology is an efficient method for increasing production and transforming oil fields with long-term water injection development and prominent preferential flow channels.

## Figures and Tables

**Table 1:** Detailed parameters of experimental cores.

Core Samples	Core Number	Experiments	Core Length/cm	Core Diameter/cm	Permeability /m D
SZ36-1-J20S1 1-006A	1	Parallel Flow Displacement Experiments	4.69	2.48	535.72
SZ36-1-J20S1 1-006A	2		4.99	2.51	21.36
SZ36-1-N12 3-027B	3		2.69	2.52	143.90
SZ36-1-N12 3-031B	4		2.72	2.51	8.89
SZ36-1-N12 3-029B	5		4.98	2.53	31.34
SZ36-1-N12 3-032B	6		5.04	2.52	6.31
SZ36-1-N12 3-028B	7		2.60	2.52	61.65
SZ36-1-J20S1 1-017A	8		2.53	2.50	31.33
Artificial Cores	9	Plugging Rate Tests	4.99	2.50	533.57
	10		5.03	2.53	142.16
	11		5.01	2.49	40.79
	12		4.87	2.50	139.70
	13		4.95	2.51	252.68
	14	Plugging Strength Tests	4.82	2.49	523.15
	15		2.69	2.51	141.37
	16		5.02	2.52	246.71

**Table 2:** Basic data.

Name	Unit	Value
Geothermal Gradient	℃/100 m	2.8
Formation Pressure Coefficient	MPa/100 m	0.97
Buried Depth	m	1600
Single Layer Thickness	m	50
Reservoir Porosity	%	27
Formation Crude Oil Viscosity	MPa·s	15

**Table 3:** Solidification morphology of TDS.

Organic Solvent Content (%)	PK01 Content (%)	PK02 Content (%)	Regulator Content (%)	P04 Content (%)	Temperature (°C)	Curing State
96.7	3	0	0.3	2	90	Partial Curing
				2.5	90	Mostly Curing
				3	90	Mostly Curing
				4	80	Mostly Curing
				5	60/70	Partial Curing/Mostly Curing
				6	50/60	Partial Curing/Mostly Curing
				7	50/60	Mostly Curing
96.4	3	0.3	0.3	2	80/90	Partial Curing/Mostly Curing (Able to sway)
				2.5	80/90	Partial Curing/Mostly Curing
				3	75/85	Partial Curing/Mostly Curing
				4	80	Fully Curing
				5	60	Fully Curing
				6	50	Fully Curing
				7	50	Fully Curing

**Table 4:** Permeability changes for each core before and after plugging.

Experimental Group Number	Core Number	Initial Permeability, P_b_/m D	Initial Permeability Max–Min Ratio/C	Permeability After Treatment, P_a_/m D	Permeability Max–Min Ratio After Treatment/C	Permeability Ratio After and Before Treatment, P_ab_/%
1	Core1	535.72	25.08	525.89	13.26	98.17
	Core2	21.36		39.65		185.63
2	Core3	143.90	16.19	137.39	6.41	95.48
	Core4	8.89		21.44		241.17
3	Core5	31.34	4.97	29.83	2.65	95.18
	Core6	6.31		11.24		178.13
4	Core7	61.65	1.97	59.50	1.2	96.51
	Core8	31.33		49.49		157.96

$P_{ab}$ ($P_{ab}=P_{a}/P_{b}$, $P_{a}$ is the permeability after treatment, $P_{b}$ is the initial permeability) is an important parameter to characterize whether the heterogeneity of the reservoir is improved or not. $P_{ab}$ greater than 1 indicates that the reservoir has been improved, $P_{ab}$ less than 1 indicates that the reservoir has been damaged, and the closer to 1, the less damage it has done.

**Table 5:** Results of the plugging rate tests.

**Core Number**	$\mathrm{Initial}\;\mathrm{Permeability},\;K_{1}$/m D	Average Permeability After Plugging, $K_{2}$/m D	Plugging Rate, *w*/%
1	533.72	10.47	98.04
2	142.16	3.39	97.62
3	40.79	1.74	95.73
4	139.70	6.98	95.00
5	252.68	12.18	95.18

**Table 6:** Slug design for IDBPRT.

Slug Name	Function and Purpose
Pre-flushing fluid slug	Wellbore cleaning, increased permeability, pre-flush formations
Disposal fluid slug	Eliminate damage to high-permeability reservoirs
Displacement fluid slug	Separating the acid from the TDS
TDS fluid slug	Plugging high-permeability reservoirs
Displacement fluid slug	Separating the acid from the TDS
Disposal fluid slug	Eliminate damage to secondary permeability reservoirs
Displacement fluid slug	Separating the acid from the TDS
TDS fluid slug	Plugging secondary permeable reservoirs
Displacement fluid slug	Separating the acid from the TDS
Disposal fluid slug	Eliminate damage to low-permeability reservoirs
……	……
Displacement fluid slug	Protecting slug and preventing TDSfrom gumming up in the wellbore

## Data Availability

The datasets presented in this article are not readily available because the data are part of an ongoing study.

## Abbreviations

The following abbreviations are used in this manuscript:

TDS	temperature-controlled diversion system
IDBPRT	Intelligent Diversion and Balanced Plugging Removal Technology
ACC	auxiliary agent concentration
$T_{\mathrm{gel}}$	gel-forming temperature
COFs	covalent organic frameworks
$P_{\mathrm{ab}}$	permeability ratio after and before treatment
$P_{a}$	the permeability after treatment
$P_{b}$	the permeability before treatment
NMR	nuclear magnetic resonance
SEM	scanning electron microscope
$\frac{V}{h_{e}}$	injection strength
V	displacement
h_e_	effective absorption thickness
L	plugging length
L′	mixed-phase band length
∅	porosity
α	safety factor
n	number of temporary plugging
